# Supplementary material for: WordCommentsAnalyzer: A windows software tool for qualitative research
Source: F1000Res. 2018 Sep 4;7:536. Originally published 2018 May 3. [Version 2] doi: 10.12688/f1000research.14819.2 (PMC6137411; doi:10.12688/f1000research.14819.2)
Supplement: Supplementary file 3 [file f1000research-7-17614-s0002.tgz › b2a79496-4ada-47c4-9682-a99ce867836d.zip!/Eight Iranian Health Journals/Journal of Health System Research _27.docx]

© Journal of Health System Research . This work is licensed under CC BY-NC 4.0 (https://creativecommons.org/licenses/by-nc/4.0/).

Volume & Issue: دوره 9، شماره 5؛ 1392

Title: پیشگیری از خودکشی در کشورهای در حال توسعه: موضوعی در اولویت

http://hsr.mui.ac.ir/index.php/jhsr/article/view/737

خودکشی به معنای خاتمه بخشیدن به زندگی با قصد و اراده می‌باشد که بر اساس شواهد موجود، در کشورهای در حال توسعه رو به افزایش است. هدف از نگارش مقاله مروری حاضر، شناخت الگوهای موجود خودکشی در این قبیل کشورها و ارایه رهیافت‌های پیشگیرانه‌ مناسب می‌باشد. در سال 1391 موتور جستجوی Pubmed (http://www.ncbi.nlm.nih.gov/pubmed) با بررسی واژگان Suicide، Prevention و Developing countries در عنوان مقالات، مورد جستجو قرار گرفت. در مرحله بعد، با تغییر در شیوه جستجو و همچنین با بهره‌گیری از منابع مورد استفاده در مقالات به دست آمده، نهایت دقت به عمل آمد تا مرتبط‌ترین و به‌روزترین مقالات، مورد استفاده این مطالعه مروری قرار گیرند. بخش عمده‌ای از افزایش خودکشی در کشورهای در حال توسعه، در بین جوانان و به ویژه زنان جوان ازدواج کرده رخ می‌دهد. متأسفانه با وجود این افزایش، توجه لازم به این پدیده در کشورهای در حال توسعه صورت نمی‌پذیرد. از آن‌ جایی که الگوی رخداد خودکشی به شدت تابع شرایط فرهنگی هر جامعه‌ای می‌باشد، با شناخت دقیق موارد رخ داده، می‌توان نسبت به تهیه و تدوین برنامه‌های پیشگیری از خودکشی همت گماشت. بر این اساس، رهیافت‌های پیشگیری از خودکشی باید از رویکردی مبتنی بر جمعیت برخوردار باشد و هر سه سطح پیشگیری را در بر گیرد.

Title: دریافت سویا و خطر سرطان پستان

http://hsr.mui.ac.ir/index.php/jhsr/article/view/744

با توجه به شیوع رو به افزایش سرطان پستان در بین نسل‌های مهاجر آسیایی به کشورهای غربی، به نظر می‌رسد که عوامل محیطی از قبیل عوامل مرتبط با سبک زندگی و رژیم غذایی، بیش از عوامل ژنتیکی در وقوع سرطان نقش داشته باشند. به منظور جستجوی مقالات در این زمینه از موتور جستجوی Pubmed استفاده گردید و کلماتی همچون Breast cancer، Soy، Isoflavone و Phytoesterogen به عنوان کلید واژه جهت جستجو انتخاب شد و مقالاتی که در بین سال‌های 1990 تا 2012 به چاپ رسیده بودند، انتخاب گردید. مطالعات تجربی متعددی نشان داده‌اند که هم سویا و هم ایزوفلاوون آن ممکن است فرد را از ابتلا به سرطان پستان محافظت نماید. با این حال، برخی مطالعات نشان داده‌اند که ایزوفلاوون سویا، تقسیم سلولی را در شرایط آزمایشگاهی افزایش می‌دهد و یا با اثربخشی تاموکسیفن تداخل دارد. در واقع، مطالعات اپیدمیولوژیک که ارتباط دریافت سویا و سرطان پستان را بررسی کرده‌اند، نتایج متفاوتی نشان داده‌اند. یافته‌ها حاکی از آنند که مصرف سویا در مقادیری که در بین زنان کشورهای آسیایی رایج است، دارای اثرات محافظتی علیه سرطان پستان است. در واقع، میزان سویای مصرفی باید به اندازه‌ای باشد که با تأمین 50-20 میلی‌گرم ایزوفلاوون در روز همراه باشد. این اثر محافظتی سویا علیه سرطان پستان، بهترین اثربخشی را دارد و پیامدش زمانی مشهودتر است که در دوران کودکی مصرف شده باشد. با این حال، مصرف سویا در نوجوانی نیز نسبت به مصرف سویا در بزرگسالی دارای مزایای بیشتری است.

Title: بررسی نقش رفتار در روابط اجتماعي بر سلامت روح و روان از دیدگاه امام علی (ع)

http://hsr.mui.ac.ir/index.php/jhsr/article/view/780

با توجه به وجود ارتباط بین نگرش‌های مذهبی و رفتار می‌توان آموزه‌های دین را برای هماهنگ کردن ابعاد شخصیت و رفتار افراد در ارتقای سلامت روح و روان به کار گرفت. بی‌تردید با شناخت صحیح مفاهیم قرآن و نهج‌البلاغه و به کارگیری تعالیم اسلامی می‌توان فرد و جامعه را برای رسیدن به سلامت روحی و روانی یاری نمود. از این رو پژوهش حاضر با هدف بررسی نقش رفتار بر سلامت روان از دیدگاه امام علی (ع) انجام گرفت. پژوهش حاضر یک مطالعه کتابخانه‌ای است که در آن سعی شد از قرآن، نهج‌البلاغه و غررالحکم استفاده گردد. در ابتدا نقش رفتار در تأمين سلامت روح و روان و مكانيزم تأثير رفتار در تأمين سلامت رواني افراد بیان گردید. سپس عوامل بهبوددهنده و آسیب‌های رفتار ناپسند در کلام امام علی (ع) ذکر شد و آثار هر کدام بر سلامت روان بیان گردید. در این مطالعه نقش رفتار بر سلامت روان از دیدگاه امام علی (ع) مورد بررسی قرار گرفت. یافته‌های تحقیق نشان داد که رفتار پسندیده از موارد مهم اثرگذار بر سلامت روان است و در ارتقای آن مؤثر می‌باشد. اسلام به روابط اجتماعي بسيار اهميت مي‌دهد؛ چرا که روابط صحيح و عمل به دستورهاي اسلام در اين بخش، در سلامتي روح و روان و تكامل افراد و كل جامعه نقش حياتي دارد. از اين رو بخش قابل توجهي از مباحث فقه به تنظيم و چگونگي روابط انسان‌ها مربوط مي‌شود. براي ايجاد رابطه مطلوب با ديگران بايد اصول و معيارهايي رعايت شود. اصول و معيارهاي رعايت حقوق ديگران به دو دسته تقسيم مي‌شود. دسته اول صفات و خصوصيات رفتاري است كه فرد در برابر ديگران بايد از خود بروز دهد و بدان‌ها آراسته شود. دسته دوم رفتارهاي ناپسندي است كه فرد بايد از آن‌ها اجتناب کند. از جمله صفات مناسب، اخلاقي است كه به بهبود روابط اجتمایی و سلامت روان كمك ‌كند.

Title: بررسی نیازهای خانواده بیماران بستری در بخش‌های مراقبت ویژه

http://hsr.mui.ac.ir/index.php/jhsr/article/view/1012

بحران ایجاد شده به دنبال بستری شدن یک عضو خانواده در بخش ویژه، موجب ایجاد نیازهای جدیدی در اعضای خانواده می‌گردد. در صورت برآورده نشدن این نیازها، استرس و اضطراب زیادی در اعضا ایجاد و باعث کاهش توانایی آن‌ها در تطابق با بحران می‌شود. هدف از این مطالعه، بررسی نیازهای خانواده‌های بیماران بستری در بخش مراقبت‌های ویژه بود. این مطالعه از نوع مروری بود و برای انجام آن، مطالعات مرتبط با زبان‌های فارسی و انگلیسی در پایگاه‌های علمی Sience direct، Pubmed، Scopus، Willy، Ovid و Springer جستجو و بررسی شدند. معیار ورود، شامل مطالعات توصیفی و کیفی بود و معیار خروج، شامل مطالعاتی بود که گروه هدف آن‌ها، خانواده بیماران اطفال و یا نوزادان در بخش‌های ویژه بود و مطالعاتی که در سایر بخش‌های ویژه، غیر از ICU (Intensive care unit)، انجام شده بودند. از 96 مطالعه یافت شده، 81 مورد حذف و 15 مطالعه انتخاب و بررسی شد. مهم‌ترین نیاز خانواده‌ها، نیاز اطلاعاتی به منظور کسب اطلاعات در مورد وضعیت بیمار و نوع درمان‌های استفاده شده برای وی بود تا از این طریق، از انجام بهترین مراقبت برای بیمارشان مطمئن شوند. نیاز رده مراقبت نیز در اکثر مطالعات در رده سوم اهمیت قرار داشت و مطالعات اندکی که آن را در رده دوم اهمیت قرار داده بودند، هدف خانواده‌ها از ملاقات بیمارشان را حمایت از بیمار خود و اطمینان از بهترین مراقبت در مورد وی بیان کرده بودند. نیازهای رده راحتی و حمایت نیز در پایین‌ترین درجه اهمیت مطرح شده بودند. نیازهای خانواده بیماران بخش ویژه، به ترتیب اهمیت شامل اطلاعات، اطمینان، مجاورت، حمایت و راحتی هستند. اگر چه هدف خانواده‌ها از دریافت اطلاعات، کسب اطمینان از این موضوع است که بیمارشان بهترین مراقبت ممکن را دریافت می‌کند، اما اطمینان پرستار از ارایه بهترین مراقبت برای بیمار کافی نیست و پرستاران باید در موقعیت‌های کلیدی جهت کمک به خانواده‌های بیماران بخش‌های مراقبت ویژه و رفع نیازهای آن‌ها بکوشند و با برقراری ارتباط صحیح با آن‌ها، اطلاعات درست در مورد وضعیت بیمار و نحوه درمان به آن‌ها بدهند و آن‌ها را از بهترین مراقبت در مورد بیمارشان مطمئن نمایند. در واقع، بیمار و خانواده وی یک واحد محسوب می‌شوند و برای دریافت مراقبت، نباید خانواده بیمار مورد غفلت واقع شود.

Title: بررسی وضعیت تغذیه و ترکیب بدن اعضای تیم‌های ورزشی دانشجویان دختر دانشگاه علوم پزشکی اصفهان

http://hsr.mui.ac.ir/index.php/jhsr/article/view/748

مقدمه: مطالعات گوناگون نشان دادهﺍند که دریافت ناکافی و نامتعادل مواد مغذی بر اجرای صحیح حرکات ورزشی تأًثیر منفی می‌گذارد و باعث کاهش کارایی جسمانی ورزشکاران می‌گردد. هدف این مطالعه بررسی وضعیت تغذیه و ترکیب بدن دختران دانشجوی ورزشکار دانشگاه علوم پزشکی اصفهان بود.  روش‌ها: این مطالعه به صورت توصیفی- مقطعی بر روی 38 دختر دانشجوی تیمﻫای ورزشی دانشگاه علوم پزشکی اصفهان انجام گردید. دادهﻫای عمومی با استفاده از پرسش‌نامهﻫای فرم اطلاعات عمومی و اندازهﮔیریﻫای تن سنجی جمع‌آوری شد و دادهﻫای دریافت غذایی به وسیله ثبت غذایی سه روزه تعیین گردید. بررسی دادهﻫا با استفاده از نرم‌افزارﻫای SPSS نسخه 16 و FPII (Food processor II) انجام گرفت. یافته‌ها: در افراد مورد بررسی با میانگین سنی 7/21 سال، میانگین دریافت انرژی، پروتئین، کربوهیدرات و چربی در حدود مقادیر توصیه شده DRI (Dietary reference intakes) بود؛ پروتئین، کربوهیدرات و چربی به ترتیب 6/14، 8/56 و 6/28 درصد از کل انرژی دریافتی روزانه را تشکیل می‌داد. میانگین دریافت فیبر (g/d 8/15) پایینﺗر از مقدار توصیه شده DRI بود. در بین ریز مغذیﻫا، میانگین دریافت پانتوتنیک اسید، فولات، کلسیم و منیزیم کمتر؛ پیرودوکسین، 12B و  روی مطابق و ویتامین‌های A، E، C، تیامین، ریبوفلاوین، نیاسین، آهن، فسفر و سلنیوم بیش از مقادیر توصیه شده DRI بود. میانگین BMI (Body mass index) 2m/kg 6/2 ± 7/20، میانگین WHR (Waist to hip ratio) 03/0 ± 74/0، میانگین TSF (Triceps skin fold) mm 7/4 ± 08/18 و میانگین چربی بدن 2/7 ± 3/25 درصد بود. نتیجه‌گیری: مشکل اصلی در رژیم غذایی ورزشکاران مورد مطالعه، دریافت فیبر پایین و کمبود دریافت پانتوتنیک اسید، فولات، کلسیم و منیزیم بود.

Title: تعیین میزان DMFT در دانش‌آموزان 11-7 ساله شیراز و ارتباط آن با میزان فلوراید در آب آشامیدنی

http://hsr.mui.ac.ir/index.php/jhsr/article/view/757

مقدمه: یکی از اهداف سازمان بهداشت جهانی (WHO یا World health organization) و فدراسیون بین‌المللی دندانپزشکی (FDI یا Federation dentaire internationale) کاهش شاخص DMFT (Decayed- missing- filled- teeth) کمتر از 1 است. توصیه این مراجع بر آن است که شاخص DMFT به طور دوره‌ای مورد بررسی قرار گیرد. هدف از انجام این طرح، تعیین شاخص DMFT در دانش‌آموزان 11-7 ساله و ارتباط آن با میزان فلوراید در آب آشامیدنی شهر شیراز بود. روش‌ها: پژوهش حاضر یک مطالعه توصیفی از نوع مقطعی- تحلیلی بود. تعداد 760 دانش‌آموز برای محاسبه شاخص DMFT در چهار ناحیه آموزش و پرورش شیراز و تعداد 75 نمونه آب جهت تعیین میزان فلوراید آن مورد بررسی قرار گرفت. نمونه‌برداری از ایستگاه‌های تأمین کننده آب شرب شیراز و آب شرب مدارس انجام گردید. فلوئور آب با روش اسپادنز و توسط دستگاه اسپکتروفتومتر مدل U2000-DR تعیین گردید. جهت تعیین DMFT ابتدا مدارس به روش خوشه‌ای و دانش‌آموزان به روش تصادفی انتخاب گردید و سپس معاینه افراد مورد مطالعه و تکمیل فرم مخصوص، توسط بهداشت‌کار دهان و دندان انجام گرفت. یافته‌ها: میانگین غلظت فلوراید آب شرب شیراز 69/0 l/mg و شاخص DMFT 935/0 بود. بیشترین و کمترین مقدار میانگین DMFT در ناحیه 4 و 1 به ترتیب برابر 35/1 و 47/0 بود. مقدار میانگین DMFT در دختران و پسران مساوی و برابر 93/0 به دست آمد. ارتباط معنی‌داری بین مقدار DMFT و گروه‌های مختلف سنی دیده نشد (05/0 < P). بیشترین مقدار M (دندان کشیده شده) مربوط به گروه سنی 8 سال با میانگین 42/2 بود. میانگین DMFT در نواحی 3 (26/1) و 4 (35/1) تفاوت معنی‌داری با میزان میانگین این شاخص در نواحی 2 (47/0) و 1 (65/0) داشت (001/0 > P). نتیجه‌گیری: مقدار شاخص DMFT در دختران و پسران در این مطالعه یکسان به دست آمد که این شاخص برابر 935/0 بود و از میانگین قبلی به دست آمده در شیراز در سال 1375 (9/2 = DMFT) بسیار کمتر شده بود. هر چند که میانگین میزان فلوئور آب شرب شیراز کمتر از حد استاندارد بود، اما با توجه به بهبود رعایت بهداشت دهان و دندان در خانواده‌ها، میانگین این شاخص در دانش‌آموزان مورد مطالعه در شیراز از 1 کمتر بود.

Title: بررسی فراوانی افسردگی و عوامل مرتبط با آن در نوبت‌کاران پالایشگاه نفت تهران

http://hsr.mui.ac.ir/index.php/jhsr/article/view/763

مقدمه: نوبت‌کاری یکی از خطرات شغلی به خصوص در بین کارگران صنایع مي‌باشد. کارگران پالایشگاه از جمله کارگران صنایعی هستند که لازم است در سیستم نوبت‌کاری کار کنند و احتمالاً با مشکلات مربوط به نوبت‌کاری از قبیل افسردگی روبه‌رو هستند. تحقیقات در صنعت پالایش نفت در مورد شیوع افسردگی در نوبت‌کاران چرخشی (Rotational shift workers) نادر است. بنابراین مطالعه حاضر با هدف تعیین فراوانی افسردگی و عوامل مرتبط با آن در نوبت‌کاران پالایشگاه نفت تهران شهید تندگویان تهران انجام شد. روش‌ها: در این مطالعه مقطعی سطح افسردگی نوبت‌کاران توسط آزمون خود اجرای افسردگی Beck (Beck depression inventory) 21 سؤالی مورد بررسی قرار گرفت. داده‌های مربوط به متغیرهای مستقل توسط پرسش‌نامه عمومی خود اجرا جمع‌آوری گردید. معیار ورود به پژوهش نوبت‌کار بودن حداقل با سابقه یک سال در پالایشگاه نفت تهران و اعلام رضایت کتبی جهت شرکت در مطالعه بود. بنابراین 307 از 355 نفر نوبت‌کار که راضی به شرکت در مطالعه بودند، وارد پژوهش شدند. معیار خروج از مطالعه عدم رضایت جهت شرکت در مطالعه بود. داده‌ها توسط نرم‌افزار آماري SPSS نسخه 16 آناليز شد. یافته‌ها: 307 نوبت‌کار مورد مطالعه به صورت چرخشی در برنامه نوبت‌کاری رو به عقب (4 شب، 3 روز استراحت، 4 عصر، 1 روز استراحت و 4 صبح) به صورت متوالی مشغول به کار بودند. گستره سنی 62-20 سال و میانگین سنی و نمره افسردگي به ترتيب 40/10 ± 49/34 سال و 25/9 ± 97/6 بود. همچنین 9/42 درصد جمعیت مورد پژوهش نمره افسردگی بیش از 10 کسب کردند. در این مطالعه بین نمره افسردگی و سن ارتباط معکوس وجود داشت (001/0 P <). در ضمن اختلاف معنی‌داری از نظر نمره افسردگی در گروه‌های مختلف فعالیت فیزیکی مشاهده گردید (001/0 P =). نتیجه‌گیری: نتایج این پژوهش نشان‌دهنده بالا بودن نسبي فراواني افسردگی در بین نوبت‌کاران پالایشگاه نفت تهران بود. همچنین نتایج نشان داد که عدم فعالیت فیزیکی منظم نیز می‌تواند یکی از عوامل ایجاد افسردگی در آنان باشد.

Title: بررسی وضعیت آلودگی صوتی در یک کشتارگاه صنعتی مرغ

http://hsr.mui.ac.ir/index.php/jhsr/article/view/767

مقدمه: امروزه مواجهه با صدا یکی از مهم‌ترین مشکلات بهداشتی کارگران صنایع می‌باشد. سر و صدا می‌تواند باعث بروز افت شنوایی و بسیاری از مشکلات دیگر شود. بنابراین رسیدگی به معضل مواجهه با صدا باید جزو اولویت‌های برنامه‌های بهداشتی صنایع قرار گیرد. این مطالعه با هدف بررسی وضعیت آلودگی صوتی و افت شنوایی ناشی از مواجهه با صدا در یک کشتارگاه مرغ انجام شد.   روش‌ها: این مطالعه از نوع توصیفی بود که به صورت موردی در تابستان 1391 در یک کشتارگاه مرغ انجام شد. با استفاده از دستگاه صداسنج مدل 1358TES که با دستگاه مدل 1356 TESکالیبره شد، شدت صوت در نقاط مختلف کشتارگاه اندازه‌گیری و ثبت شد. سپس داده‌های حاصل با استفاده از نرم‌افزار SPSS نسخه 18 و آزمون آماری One sample t مورد تجزیه و تحلیل قرار گرفت.   سنجش‌های به عمل آمده شامل تراز فشار صوت Sound pressure level (SPL) صداهای کوبه‌ای و آنالیز فرکانسی نقاطی بود که از سر و صدای بیش از حد مجاز برخوردار بودند. سنجش‌ها و اندازه‌گیری‌های به عمل آمده نشان داد که آلودگی صوتی در برخی از این ایستگاه‌ها بالاتر از حد مجاز بود.

Title: تأثیر هشت هفته تمرینات اصلاحی منتخب بر اختلالات اسکلتی- عضلانی مرتبط با کار معلمان شهر اصفهان با مداخله ارگونومی

http://hsr.mui.ac.ir/index.php/jhsr/article/view/787

مقدمه: اختلالات عضلاني- اسکلتي مرتبط با کار به عنوان يکي از مشکلات سلامتي عمومي در سراسر دنيا و علت اصلي ناتوانايي‌ها به شمار مي‌رود. هدف از تحقيق حاضر، بررسي شيوع اختلالات عضلاني- اسکلتي مرتبط با کار معلمان شهر اصفهان و تأثير 8 هفته تمرينات اصلاحي منتخب و مداخلات ارگونومي بر آن‌ها بود. روش‌ها: برای این امر از پرسش‌نامه Nordic و روش ارزيابي سريع ريسک فاکتور Quick exposure check (QEC) استفاده شد. نتایج با استفاده از روش آماری SPSS مورد تجزیه و تحلیل قرار گرفت. یافته‌ها: تفاوت معنی‌داری بین نواحی مختلف بدن در خصوص اختلالات عضلانی- اسکلتی مشاهده شد (05/0 > P). در نواحي کمر (2/62 درصد)، گردن (4/47)، شانه (2/45 درصد)، زانو (2/42 درصد) و پا/قوزک پا (8/37 درصد) بيشتر بود. مچ/دست (1/36 درصد)، باسن- ران (5/23 درصد)، نواحي پشت (9/13 درصد) و آرنج (3/4 درصد) در مراحل بعدي قرار گرفتند. بر اساس نتايج QEC، 75 درصد از وظايف مورد مطالعه معلمان در سطح ریسک خطر بالا قرار داشت. همچنين امتياز QEC نشان داد که نوشتن و تدريس روي تابلوي کلاس بالاترين امتياز را در ميان وظايف به خود اختصاص داد (63/57 درصد). به عبارت ديگر از نظر سطح ريسک در سطح بالايي قرار داشت و از نظر ارزيابي، اقدام‌هاي اصلاحي مي‌بايست اعمال شود. به دنبال 8 هفته تمرينات اصلاحي و مداخلات ارگونومي ميزان اختلالات کمر، شانه، گردن و دست/مچ بهبودي معني‌داري مشاهده شد (05/0 > P)، به طوري که در نواحي گردن (70 درصد)، کمر (6/66 درصد)، شانه (5/33 درصد) و مچ دست (50 درصد) معلمان کاهش قابل توجهی در درد مشاهده شد. نتیجه‌گیری: نتایج نشان داد که ميزان شيوع اختلالات عضلاني- اسکلتي در معلمان مورد مطالعه به نسبت بالا بود. علاوه بر آن بالا بودن سطح ريسک خطر نشانه آسيب‌زا بودن شرايط و محيط کار در مدارس بود. اقدامات اصلاحي اعمال شده در معلمان موجب کاهش اختلالات عضلاني- اسکلتي شد. از این رو اصلاح وضعيت پوسچر در تکنيک تدريس روي تابلوي کلاس توصيه مي‌گردد.

Title: ارتباط خودكارامدي عمومي و حمایت اجتماعی درک‌شده با کیفیت زندگی بیماران قلبی- عروقی: یک مطالعه مقطعی

http://hsr.mui.ac.ir/index.php/jhsr/article/view/1053

مقدمه: بيماري‌هاي قلبي- عروقي به صورت يك فاكتور مستقل روي كيفيت زندگي بيماران تأثير منفي بسزايي بر جای مي‌گذارد. با این وجود افزایش خودکارامدی عمومی و حمایت اجتماعی درک‌شده می‌تواند در سازگاری بهتر بیماران با عوارض ناشی از بیماری و بهبود کیفیت زندگی آنان مؤثر باشد. از این رو این مطالعه با هدف بررسی تأثیر خودكارامدي عمومي و حمایت اجتماعی درک‌شده بر کیفیت زندگی بیماران قلبی- عروقی انجام شد.  روش‌ها: در یک مطالعه مقطعي 116 بيمار قلبي- عروقي بستري شده در مراکز بيمارستانی شهر ایلام با روش نمونه‌گيري در دسترس انتخاب و مورد بررسی قرار گرفتند. در این مطالعه پرسش‌نامه‌‌های كيفيت زندگي ( World Health Organization Quality of Lifeیا (WHOQOL-BREF، خودكارامدي عمومي و حمايت اجتماعي درك‌شده مورد استفاده قرار گرفت. اطلاعات مطالعه پس از جمع‌آوری و ورود به نرم‌افزار SPSS نسخه 21 و در سطح معنی‌داری 05/0 = α مورد تجزیه و تحلیل قرار گرفت. یافته‌ها: 3/35 درصد شركت‌كنندگان در مطالعه مرد و 7/64 درصد زن بودند که 8/82 درصد آنان متأهل و 2/17 درصد مجرد بودند. 6/2 درصد از بیماران کیفیت زندگی کنونی خود را بسیار بد، 4/3 درصد بد، 3/29 درصد متوسط، 9/50 درصد خوب و 8/13 درصد کیفیت زندگی خود را بسیار خوب ارزیابی نمودند. همچنین 3/4 درصد افراد از سلامت کنونی خود خیلی ناراضی، 2/11 درصد ناراضی، 2/32 درصد نه راضی و نه ناراضی، 8/44 درصد راضی و 5/9 درصد خیلی راضی بودند. میانگین نمره کیفیت زندگی بیماران در ابعاد سلامت جسمانی 32/3 ± 18/22، سلامت محیطی 08/5 ± 76/25، سلامت روانی 79/4 ± 94/19 و روابط اجتماعی 26/3 ± 79/10 به دست آمد. همچنین میانگین نمره خودکارامدی عمومی 97/26 و حمایت اجتماعی 02/25 بود. همبستگی معنی‌داری بین خودکارامدی عمومی و حمایت اجتماعی درک‌شده با ابعاد کیفیت زندگی وجود داشت، به طوری که با افزایش میزان خودکارامدی عمومی و حمایت اجتماعی درک‌شده، میانگین نمره سلامت جسمانی، سلامت محیطی، سلامت روانی و روابط اجتماعی افزایش می‌یافت (05/0 < P). نتیجه‌گیری: خودکارامدی عمومی و حمایت اجتماعی درک‌شده تأثیر بسزایی در افزایش کیفیت زندگی داشت. از این رو طراحی و اجرای مداخلات آموزشی برای افزایش حمایت اجتماعی و خودکارامدی عمومی و همچنین کیفیت زندگی در بیماران قلبی- عروقی توصیه می‌گردد.

Title: بررسی سواد سلامت بزرگسالان شهر اصفهان

http://hsr.mui.ac.ir/index.php/jhsr/article/view/1221

مقدمه: دسترسی به اطلاعات مرتبط با سلامتی و آگاهی یافتن از مسایل بهداشتی و بیماری، فاکتور مهم و تعیین‌کننده سلامتی است. انتظار می‌رود که افراد برای داشتن سطح بالای سلامتی از این گونه اطلاعات استفاده نمایند. این نقش فعال بیماران، نیازمند دارا بودن سطح بالایی از سواد سلامت می‌باشد. در این مطالعه با استفاده از دو ابزار Short test of functional health literacy in adults (S-TOFHLA) و Newest vital signe (NVS) سواد سلامت در بزرگسالان شهر اصفهان مورد ارزیابی قرار گرفت. روش‌ها:در مطالعه‌ای مقطعی به تعداد 525 نفر از افراد بزرگسال (بالای 18 سال) شهر اصفهان مورد بررسی قرار گرفتند. برای جمع‌آوری داده‌ها به طور همزمان از دو پرسش‌نامه S-TOFHLA و NVS پس از اطمینان از اعتبار ترجمه و تعیین روایی و پایایی آن‌ها استفاده شد. داده‌های جمع‌آوری شده به کمک نرم‌افزار SPSS نسخه 18مورد تجزیه و تحلیل قرار گرفت. یافته‌ها: در این پژوهش به تعداد 525 نفر بزرگسال (18 سال به بالا) با میانگین سنی 5/12 ± 6/33 سال وارد مطالعه شدند. بر اساس پرسش‌نامه S-TOFHLA 5/46 درصد افراد سواد سلامت کافی، 38 درصد سواد سلامت مرزی و 5/15 درصد افراد سواد سلامت ناکافی داشتند. پرسش‌نامه NVS نیز نشان داد که 38 درصد از افراد سواد سلامت کافی، 6/36 درصد سواد سلامت مرزی و 4/25 درصد سواد سلامت ناکافی داشتند. ضریب همبستگی Pearson با استفاده از نرم‌افزار آماری SPSS نشان داد که بین نمرات دو پرسش‌نامه S-TOFHLA و NVS همبستگی بالایی (72/0 = r) وجود داشت و به لحاظ آماری معنی‌دار بود (001/0 < P). در این مطالعه بین سطح سواد سلامت و سن، سطح تحصیلات، جنس و وضعیت اقتصادی ارتباط معنی‌دار آماری مشاهده شد، به طوری که سواد سلامت ناکافی در افراد با سنین بالاتر، تحصیلات کمتر، زنان و قشر کم درامد شایع‌تر بود. نتیجه‌گیری: نتایج این پژوهش نشان داد که دو پرسش‌نامه S-TOFHLA و NVS برای به کارگیری در جامعه بزرگسالان فارسی زبان در جمعیت مورد مطالعه از کارایی و اعتبار کافی برخوردار است. بر اساس دو پرسش‌نامه مذکور طیف وسیعی از بزرگسالان شهر اصفهان سواد سلامت ناکافی و مرزی داشتند كه اين خود لزوم توجه بيشتر به امر سواد سلامت در برنامه‌هاي ارتقاي سلامت را مي‌رساند.

Title: اندازه‌گیری و تحلیل راهکارهای كاهش زمان انتظار مراجعين به مراكز درماني عمومی بر پایه‌ شبيه‌سازي

http://hsr.mui.ac.ir/index.php/jhsr/article/view/1335

مقدمه: امروزه، زندگی شهر نشینی و گذار بیماری‌ها، مراکز بهداشتی- درمانی را برای ارایه خدمات به چالش جدی واداشته است. مشکلی که مدیریت در این مراکز با آن مواجه است، زمان انتظار طولانی بیماران می‌باشد. زمان دریافت خدمت توسط بیمار، بسیار کمتر از زمان انتظار است. افراد برای دوری جستن از زمان‌های انتظار طولانی، به مراکز خصوصی درمان مراجعه می‌کنند. این بررسی، به منظور تعیین راهکارهای کاهش زمان انتظار مراجعین بر پایه شبیه‌سازی رخدادهای گسسته انجام شد. روش‌ها: این پژوهش به صورت مقطعي در درمانگاه عمومي انجام گرفت و با استفاده از شبيه‌سازي رخدادهاي گسسته، جريان بيمار مدل‌سازي و بهينه‌سازی شد. در ورود افراد به سيستم، اطلاعات دموگرافيک آن‌ها ثبت شد. فرم‌ها به دفترچه درماني فرد ضميمه شد و با مراجعه فرد به قسمت‌هاي مختلف، اطلاعات مربوط به زمان ورود، زمان انتظار افراد در سيستم، زمان دریافت خدمت توسط آن‌ها و زمان خروج از سيستم ثبت گردید. تجزیه و تحلیل داده‌ها با استفاده از نرم‌افزار SPSS، تعیین تابع توزیع احتمال ورود افراد به سیستم و زمان دریافت خدمت توسط آن‌ها، با استفاده از نرم‌افزار Professional easy fit و شبیه‌سازی سیستم درمانی با به کارگیری نرم‌افزار Any logic صورت گرفت. یافته‌ها: تعداد کل نمونه‌ها 400 نفر بود که بیش از 60 درصد خانم‌ها و کمتر از 40 درصد آقایان بودند.20 درصد مراجعین قبل از شروع به کار سیستم مراجعه می‌کردند که باعث اتلاف 2017 دقیقه می‌شد. زمان دسترسی به اولین خدمت از 1 تا 250 دقیقه متفاوت و به طور میانگین 59 دقیقه بود. مجموع زمان ارایه خدمت به افراد از 1 تا 20 دقیقه متغیر و میانگین آن 5/7 دقیقه به دست آمد. تأخیر در ورود پزشکان 15 دقیقه بود. زمان ورود مراجعین از تابع توزیع احتمال نمایی و زمان ارایه خدمت از تابع توزیع گاما تبعیت می‌کرد.  نتیجه‌گیری: 65 درصد مراجعین بیش از 30 دقیقه برای دریافت اولین سرویس منتظر بودند. برای کاهش زمان انتظار، 7 سناریو پیشنهاد گردید که با نرم‌افزار Any logic شبیه‌سازی شد و از بین آن‌ها، سناریوی ترکیبی تغییر زمان شروع به کار سیستم و پذیرش 30 درصد مراجعین به صورت Book appointment بهترین نتیجه را به همراه داشت.
